# Behavioural Traits in *Bos taurus* Cattle, Their Heritability, Potential Genetic Markers, and Associations with Production Traits

**DOI:** 10.3390/ani12192602

**Published:** 2022-09-28

**Authors:** Frances Margaret Titterington, Rachel Knox, Steven James Morrison, Masoud Shirali

**Affiliations:** 1 Agri-Food and Biosciences Institute, Large Park, Hillsborough BT26 6DR, UK; 2 AgriSearch, Innovation Centre, Large Park, Hillsborough BT26 6DR, UK

**Keywords:** behavioural traits, genetics, cattle, production, breeding

## Abstract

**Simple Summary:**

Cattle have the potential to seriously injure humans and cause damage to property. The risk of cattle reacting in a dangerous manner can be reduced through genetic selection for cattle which have a better temperament. A literature search was undertaken which returned papers which met the criteria of “Bovine”, “Genetics” and “Behaviour” or terms therein. Behavioural traits were grouped and their heritability, genomic associations and correlations with production traits examined. It was found that heritability estimates were more accurate in studies with large populations (*n* > 1000). Gene associations with behavioural traits were found on all chromosomes except for chromosome 13, with associated SNPs reported on all chromosomes except 5, 13, 17, 18 and 23. Generally, it was found that correlations between behaviour and production traits were low or negligible, suggesting that genetic improvement can be undertaken without negatively affecting production. There was variation between the results of the studies examined, and this underlines that any genetic study is population specific. Thus, to assess the heritability, genetic associations with production and genomic areas of interest for behavioural traits, a large-scale study of the population of interest would be required.

**Abstract:**

People who work with cattle are at severe risk of serious injury due to the size and strength of the cattle. This risk can be minimised by breeding less dangerous cattle, which have a more favourable reaction to humans. This study provides a systematic review of literature pertaining to cattle genetics relating to behaviour. The review protocol was developed using the Preferred Reporting Items for Systematic reviews and Meta-Analyses (PRISMA) framework, with Population, Exposure and Outcome components identified as Bovine, Genetics and Behaviour respectively. Forty-nine studies were identified in the sifting and assigned non-exclusively to groups of heritability (22), genomic associations (13) and production traits related to behaviour (24). Behavioural traits were clustered into the following groups: “temperament, disposition and/ or docility”, “aggression”, “chute score”, “flight speed”, “milking temperament”, “non-restrained methods” and “restrained methods”. Fourteen papers reported high accuracy (Standard Error ≤ 0.05) estimates of heritability, the majority (*n* = 12) of these studies measured over 1000 animals. The heritability estimates were found to vary between studies. Gene associations with behavioural traits were found on all chromosomes except for chromosome 13, with associated SNPs reported on all chromosomes except 5, 13, 17, 18 and 23. Generally, it was found that correlations between behaviour and production traits were low or negligible. These studies suggest that additive improvement of behavioural traits in cattle is possible and would not negatively impact performance. However, the variation between studies demonstrates that the genetic relationships are population specific. Thus, to assess the heritability, genetic associations with production and genomic areas of interest for behavioural traits, a large-scale study of the population of interest would be required.

## 1. Introduction

Cattle were domesticated around 10,000 years ago [1], and since then they have been managed by humans to derive resources and services. Cattle must attain certain performance parameters or key performance indicators (KPIs) to be useful to humans. Growth is important for cattle destined for the food chain to ensure that adequate carcass weights are reached for processing and for growing heifers to reach growth targets for calving at optimum body size [2]. Cattle produced for beef must be of sufficient quality, with consumer demand and perception of eating quality directly affecting beef profitability [3,4]. Fertility is a highly important trait in cow-calf production and profitability, and also in the dairy industry to ensure a cow will produce milk. Management of cattle to achieve these KPIs necessitates a human–animal interaction. Cattle are relatively large animals with the potential to cause serious injury to humans [5,6]. Handler safety can be impacted by a range of factors the human involved, environmental conditions, and the animal involved [7]. The animal’s reaction to a human, defined as temperament [8], is an important factor in handler safety.

Natural selection and human-directed breeding strategies have led to the development of phenotypically distinct breeds adapted to specific breeding goals. Genetic improvements cannot happen in an individual animal’s lifetime but have the advantage of being additive over subsequent generations. Genetic selection in cattle has often focused on production KPIs, which are easier to evaluate than behavioural traits which have subjective measurement and variation in how they are defined [9]. Animal temperament is a quantitative trait controlled by an animal’s genetic predisposition for behaviour. There is a range of traits indicative of an animal’s behaviour, and thus a range of measurements which can be assessed through restrained or non-restrained methods [10]. Restrained techniques monitor behaviour when the animal is restricted and non-restrained monitor behaviour when they have the ability to move freely [11]. Behavioural traits including handling, flight speed, chute test, docility test milking temperament have been described in a previous review [12], with a wide range of measurements favouring different management systems [10].

This study reviews the genetic aspect of cattle behaviour and will examine three areas of interest: heritability of temperament traits, correlation of temperament traits with production traits, and finally genomic aspects of behavioural traits. This review focuses on contemporary papers published since the year 2000 and cattle of *Bos taurus* species, which are the most populous cattle type in Europe. The objective of this paper is to summarise exciting scientific evidence for researchers, breeders, policymakers and farmers to direct breeding goals; with an aim to ultimately improve the behaviour of cattle, which in turn can improve handler safety.

## 2. Materials and Methods

### 2.1. Search Strategy

The Preferred reporting items for the systematic reviews and meta-analyses (PRISMA) framework [13] was used to design a search protocol. Following preliminary searches of available literature, suitable search categories for the Population, Exposure and Outcome (PEO) components of the research question [14], were discussed and agreed upon by the authors. The population was defined as “Bovine”, with an exposure of “Genetics” and the outcome of “Temperament”. The search algorithm was designed to return at least one of each PEO component (Table 1). PubAg, Web of Science and the ‘EBSCO Academic search elite’ option within the research database of EBSCO were queried. Date published was restricted to between January 2000 and the search date (June 2021). Two researchers conducted searches concurrently and independently, results were then cross-referenced for validation.

### 2.2. Assessment and Selection of Papers Returned by the Search

Discarded papers were sifted into four categories: ‘Non *Bos taurus’*, where the subject was *Bos indicus* or an animal of a different species; ‘cattle health and welfare’, referring to cattle disease or temperament with no genetic aspect; ‘food safety and supply’, meat quality, food supply chain and spoilage; ‘production and breeding’, genetic studies with no temperament aspect or focusing on animal management. Narrative reviews which had no original research were also excluded from the search criteria. Selected papers were sifted into three categories: heritability of temperament traits; correlation of temperament traits with production traits; and molecular/ genomic areas related to temperament traits. The categories were not mutually exclusive.

### 2.3. Synthesis of Results

Articles for inclusion in the review were independently appraised by two authors to ensure suitability and assigned to one or more categories as necessary. Once category allocation had been discussed and agreed upon by both authors, the data from the heritability and genomic association studies were tabulated. Due to the variation in statistical methods used by the authors of the different studies to assess correlation and association between production and behavioural traits, this information could not be directly compared. The relationships were categorised as favourable or non-favourable and the strength of the relationship was derived from the description given of the relationship in each study by the original authors and, where available, the correlation coefficient. The heterogeneous mix of breeds and animal types which were reported in the genomic association studies meant that a meta-analysis was not possible.

## 3. Results and Discussion

### 3.1. Study Selection

The search criteria returned a total of 2211 papers once duplicates were removed. The majority of these papers were rejected during screening. The main reason for discarding a paper was that the subject was not *Bos taurus*, including *Bos indicus* cattle and other non-bovine species. Some studies were related to cattle welfare and health, and included behaviour but no production, conversely, cattle production and breeding studies focused on the animals’ KPIs and did not record any behaviour traits. The inclusion in the search protocol of “beef” and “dairy” led to food safety and production studies which did not include the animal stage of production being returned by the protocol.

Twenty-six review papers were removed, demonstrating that this is an area of great scientific interest. The reviews were examined to ensure the current was necessary and had not been undertaken previously. It was found that 17 of the reviews were carried out before 2016, and as such were considered outdated. The nine reviews which were carried out in the most recent five-year period did not fulfil the aims of this study, some were general reviews of domestic animals and not specifically focused on cattle [15,16,17]. Of those focused on cattle, one was a general narrative review which did not collate data [18]; those which collated data focused on production traits [19], or health and welfare traits [20,21]. One reviewed novel technology and potential new measurements of temperament rather than the genetic aspects of temperament [22]. The other review was conducted in a language other than English [23] and discarded. Of the 17 reviews carried out prior to 2016, two aligned with the aims of this review [9,12]. These reviews investigated the heritability and molecular background of temperament traits and their genetic correlations with other traits. However, 34 of the studies selected by the search strategy of the current paper were published after these reviews were carried out, thus continuation of the current review was considered appropriate.

**Figure 1:**
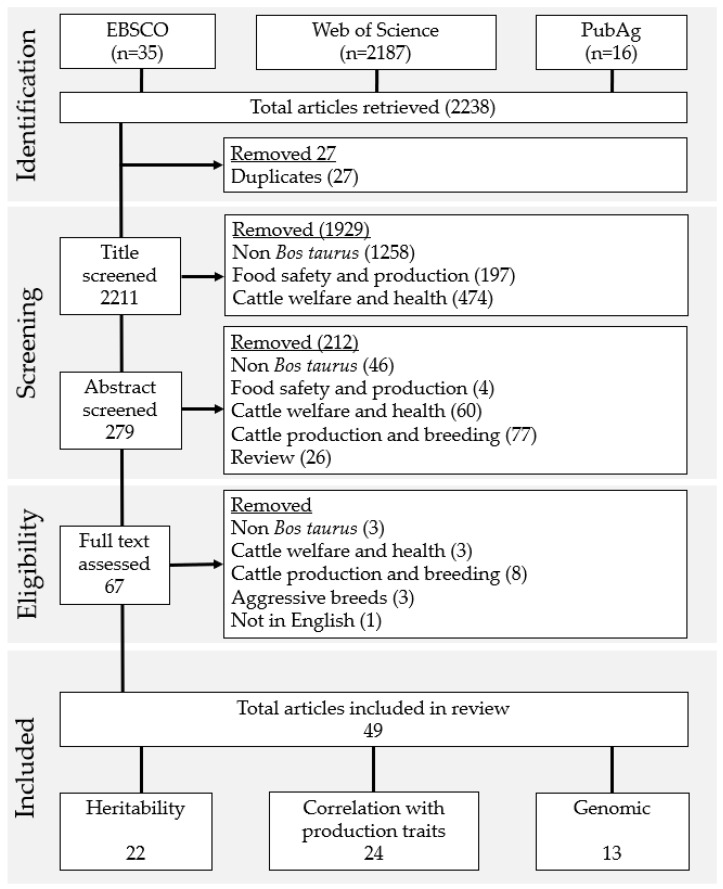
Selection process of studies returned through search protocol using PRISMA Framework [13] (Please note that the included categories were non-exclusive and the same study may have been included in multiple categories).

Sixty-seven full texts were assessed. One non-English language text was discarded [24]. Fourteen papers were allocated into the aforementioned categories and three were allocated to a category of “Aggressive breeds”. These papers were focused on aggressive breeds bred for bullfighting rather than production and did not quantify behaviour but surmised it by breed and selective SNP sweeps [25,26,27,28,29,30,31]. The 49 remaining texts were allocated into one or more of the review categories and the results were collated. Temperament traits included in each study were tabulated to compare the measurement taken and the method of assessment. Preferred Reporting Items for Systematic Reviews and Meta-Analysis (PRISMA) [13] diagram depicting the search process is presented in Figure 1.

### 3.2. Behavioural Traits

The traits found in this review are listed in Supplementary Table S1. It is difficult to directly compare traits between studies due to the variation in nomenclature and how the traits were assessed and subsequently measured. For the purposes of this study, they are grouped into eight categories: Temperament, disposition and or docility; Aggression; Chute score; Flight score; Milking temperament; Non-restrained measures and restrained measures. Forty one of the traits were reported to have been measured subjectively. Subjective measurement has been found to be repeatable and reliable within a study [32], however, without observing multiple studies and comparing the results it cannot be guaranteed that results will be reliable across multiple studies. Similarly, objective measures of behaviour differed between studies. This range of methods and measurements for behavioural traits demonstrate the difficulty in directly comparing different studies, and care must be taken when reviewing the papers.

Many of the studies tested the behavioural traits over two periods. This allowed the animal’s habituation to different testing methods to be assessed. Habituation is important as an animal’s reactions to humans can be improved through positive experiences. Furthermore, repeating the behavioural tests allows comparison at different ages, if there is a high correlation between the same trait when measured at different ages, it can be surmised that the same set of genes controls the trait at the ages recorded [33,34].

#### 3.2.1. Temperament, Disposition and/or Docility

The most reported trait was temperament, this was generally a subjectively measured trait, however, some objective measures were included such as movement in a weighing device [35] or a combination of other traits such as pen score and flight speed [36]. Subjective scoring was on a scale of 1 to 5 [36,37,38] or 1 to 9 [39,40]. In one study, the scale was converted to a binary Temperament Grouping of ‘calm’ (score 1 or 2) and ‘restless’ (scores 3, 4, or 5) [38]. Some studies did not detail the precise measurements but rather took temperament measures as assessed by expert breeders [41] or farmer assessment [42,43,44,45]. When comparing farmer-assessed temperament and handling temperament assessed by a qualified classifier were found to have a high correlation of 0.84 ± 0.06, suggesting they were similar traits [44]. Temperament was assessed at different stages of the animal’s life, for example, weaning [35] or during and before handling [37] or under different management systems such as automatic milking systems (AMS) or conventional milking systems.

The subjective nature of not only the measurement but of the nomenclature of assessing behavioural traits is demonstrated by a study which reports the temperament of cattle [37] citing a method used to report docility [46]. Docility was generally given a subjective score [41,47,48,49], however, this could be further modelled to include maternal and environmental effects [48]. Modelling multiple traits to calculate docility was a technique used by Phocas et al., 2006 who reported docility as a linear combination of aggression and escape attempts from an enclosed area [50].

#### 3.2.2. Aggression

The studies examined reported aggression to be a subjective trait which assessed either how the animal interacted with other cattle in the herd [51] or humans [50]. Aggression was scored at different times during the animals’ life, with one study assessing aggression at both parturition and during gestation [51]. Aggression was subjectively scored by farmers [42,43,51] or trained handlers [50].

#### 3.2.3. Chute Score (CS)

This review will use the term ‘Chute score’ (CS), however, the terms chute and crush are interchangeable and generally reflect the terms used in the geographic region where the study was undertaken. Chute score was generally measured subjectively with observers scoring an animal’s reaction when restrained in a chute [52,53,54,55,56,57,58]. One study implemented an ‘Objective chute score’ which was calculated as the SD of the animal’s weights collected at 5 Hz for 10 s whilst restrained in a chute [57]. The time taken to observe the animal’s behaviour was a trade-off between the time taken to record a response and the time available to record all animals [57], this varied between studies and ranged from 5 s [55] to 20 s [54]. Chute scores were on either subjective scores of 1 to 5 [53,55,57], 1 to 6 [52,54] with each increment increasing aggression. The variation in scoring scales and methods means that it is difficult to directly compare the CS reported in different studies.

#### 3.2.4. Flight Speed (FS)

Flight speed (FS) and exit velocity (EV) both describe the behaviour of an animal leaving a crush or squeeze chute. One study assessed this subjectively on a scale of 1 to 4, with the animal judged to have walked, trotted, ran or jumped, respectively, [53]. Most studies assessed this objectively by recording the time taken for an animal to traverse a set distance with the time elapsed for the animal to travel the distance between beams converted to m/s [33,55,57,59,60,61,62]. Where stated, the distance traversed ranged from 1.68 m [57] to 4.318 m [59,60]. This variation in distance means that if the animal did not travel at a constant speed over an extended distance the estimates of FS will not be directly comparable between studies.

#### 3.2.5. Milking Temperament

Five studies reported on the milking temperament or actions whilst milking of cows, with two studies using the same dataset [42,43]. Three studies subjectively measured milking temperament. Scales used varied from one to four [42,43] or one to five [63,64]. In each case, a higher score indicated a calmer animal. Although not reported as ‘Milking temperament’, one study recorded the actions of a cow during milking by counting the number of flinch steps (where the animal flinched but the foot moved no higher than the udder), flinch steps and kicks (where the foot went higher than udder), and the sum of these scores to monitor the animal’s behaviour during milking [65].

#### 3.2.6. Non-Restrained Behavioural Measures

Non-restrained measures of temperament were recorded when the animal was in an enclosed pen but was able to move freely. An animal’s reactivity can be assessed by its reaction to novel objects or humans [66] where the animals’ reactivity to an unfamiliar object is monitored. The novel object may be a human the animal is not familiar with [61,65,67] or an object [66,67]. Reaction to a novel object or human could be assessed on a subjective scale [61,67], the time spent within a certain distance of the object [65,68] or by recording the animal behaviours (such as exploration, jumping, vocalisations) and performing a Principle components analysis [66,69].

#### 3.2.7. Restrained Behavioural Measures

Restrained behaviours included the animal’s reaction when tethered [58,70], avoidance distance when restrained at the feeding barrier [69,71] or avoidance distance during weighing [34,58]. Weighing tests were assessed either subjectively on a scale of 1 (calm) to 5 (excited) or by counting movements in the weigh crate [34]. When counting the movements of the animal in the weigh crate, the movements could be classified as “rush movements” if they were vigorous. The movements in the weigh crate were further categorised by whether a human was in the animals’ sight line or not. The number of movements recorded was reported both on a linear and a categorical scale. Race score recorded on a scale of 1 (calmest) to 5 assessed animal behaviour whilst traversing a race [54]. Although the animal was not tightly restrained, this could not be considered an unrestrained test as the animals’ choice of movement was limited.

### 3.3. Heritability

Twenty-two papers which reported the heritability of the traits of interest in this review were returned in the search. This included a range of different breeds and composite breeds of both dairy and beef origin. Estimates of heritability were found to vary for each behavioural trait. Some of the variations in the estimated heritability can be explained by the variation in measuring traits previously described. However, the studies also varied by the numbers and types of cattle included. Heritability is a population parameter; therefore, reported heritability of a trait is dependent on several factors such as the experimental design, the model used for estimation, number of sampled animals, population size, breed of animals, genetic variation within animals used in the study and environmental factors. As a result, the estimated heritability will vary between studies. However, the estimated heritability can be constant across populations and species in some traits [72].

Heritability is a parameter expressing the proportion of phenotypic variation explaining genetic factors in the population [72]. The heritability of a trait ranges from 0 (no heritability) to 1, and for ease of understanding can be categorised as low (<0.15) or high (>0.40) [73]. Values between these thresholds are considered moderate heritability. Another important parameter in comparing different studies is the standard error (SE) of estimated heritability. Studies with a higher population of cattle sampled generally had lower SE [38,41,42,44,48,74] and this indicates a more accurate estimate of heritability. In this study, an SE > 0.05 was considered to indicate an inaccurate estimate of heritability. Studies which reported an SE ≤ 0.05 are listed in Table 2, with those with less accurate estimates of heritability are listed in Supplementary Table S2. Most studies which reported higher accuracy estimates of heritability had ≥843 cattle in the study, only one had fewer animals than this and although the accuracy was high, the heritability was reported as 0 [37]. Contemporary estimates of heritability from this study had a lower accuracy. Many of the studies reported heritability in addition to production traits or molecular analyses, since heritability was not the main focus of these studies the SE was not reported thus the accuracy cannot be judged.

#### 3.3.1. Heritability of Temperament Disposition and Docility

Estimates of heritability of temperament varied, ranging from low heritability for cows milked by an AMS [74] to moderate for dairy heifers [75]. A study of Brown Swiss cows reported a higher heritability of 0.38 ± 0.07, however, this had a lower accuracy [42]. Similarly, the reported heritability in docility varied from low (0.10 ± 0.01) [44] to moderate (0.34 ± 0.01) [48]. A study of docility during handling in German Simmental and German Angus calves reported high heritabilities of 0.55 ± 0.15 and 0.61 ± 0.17 respectively. However, this was a small-scale study of fewer than 150 cattle. It is notable in this study that the heritability of docility before handling was less heritable 0.13–0.17 ± ≥12. This study referred to the assessment of docility as a docility test but awarded a temperament score, demonstrating how the two terms have been used interchangeably.

There is also a notable variation in estimated heritability observed between different breeds of beef cows [37]. This is supported by the differences between differences reported by other studies, with heritability of docility varying between Angus and Limousin [49] and Pirenaica [41] cattle. This variation can be attributed to innate breed differences and variation in measures and calculation of temperament and /or docility. For example, six different models to calculate docility in Limousin cattle were used in one study from the same docility score dataset [48]. The first model only included random direct genetic effects, with the remaining five models including random direct genetic effects with a combination of maternal genetic effects, direct maternal genetic covariance of phenotypic variance, and maternal permanent environmental variance. Direct heritability estimates were moderate, ranging between 0.29 ± 0.02 (when direct maternal genetic covariance of phenotypic variance effects were dropped from the model) and 0.38 ± 0.03 (when the model included all effects). Similarly, ‘farmer-assessed temperament’ and ‘handling temperament assessed by a qualified classifier’ are reported to have varying heritability [44] (Table 2).

The importance of external environmental effects on the heritability of temperament was demonstrated by allocating embryo transfer full siblings to non-related dams, thus removing the confounding effect of dam behaviour [35]. This study reported a higher heritability than contemporary studies, with a heritability of 0.36 (no SE reported) for temperament at weaning.

#### 3.3.2. Heritability of Aggression

Four studies reported the heritability of aggression, all estimates were low, with the highest estimate of heritability for aggressiveness at parturition. Aggression could be measured at different events or stages in the animal’s life, one study reported the estimated a low heritability of aggressiveness during gestation and a moderate heritability of aggression during parturition [45]. In this study, it was reported that the animals were scored for aggression once, but it is not clear if this meant one score was recorded for behaviour throughout gestation/parturition or if the animal was assessed once. Assessing aggression over a long period of time (gestation) compared with a short period of time (parturition) means it is possible that episodes of aggression may have gone unrecorded, thus reducing the animal’s aggression score. One study which had a small number of animals (140) had a high accuracy estimate of heritability but the heritability reported was negligible [37].

#### 3.3.3. Heritability of Chute Score

Direct heritability of CS in a study of American Hereford cattle had a moderate heritability [52]. This was the only estimate of CS with a high accuracy (Table 2). Heritability of CS was found to vary by breed in a study of German Angus, Charolais Hereford and Limousin, with Limousin the least heritable (0.11 ± 0.08) and Hereford the most (0.33 ± 0.10) [53]. It is notable that the numbers sampled in this study were low, ranging from 424 to 706.

#### 3.3.4. Heritability of Flight Speed

No estimated heritability for FS was considered accurate. Variation between breeds for FS was reported in the same study, ranging from 0.11 ± 0.07 in Limousin to moderate in Charolais, German Simmental and Hereford (0.25–0.36 ± ≤0.08) [53] using a subjective scale. A much higher heritability of 0.49 ± 0.18 was reported [62], however, the large SE reflects that this was a small study of 302 cattle. One large-scale study with more than 1000 cattle reported moderate heritabilities of 0.34 ± 0.11 [60]. This study included multiple breeds and did not distinguish between breeds for the FS analysis, thus the high variation reported in FS may be a breed effect.

#### 3.3.5. Heritability of Milking Temperament

Heritability of milking temperament was reported to be low (Table 2). It is shown to vary between breeds, however, no assertions can be made as only two datasets reported high accuracy heritability of milking temperament [42,43,63].

#### 3.3.6. Heritability of Non-Restrained Behavioural Measures

No studies were returned which assessed non restrained behavioural measures for heritability.

#### 3.3.7. Heritability of Restrained Behavioural Measures

No highly accurate heritability estimates were found for restrained measures of behaviour. Only one study in the restrained behavioural measures had more than 1000 cattle [34], which recorded the time spent moving when restrained in a weighing crate. This was found to vary within the same study (ranging from 0.11 ± 0.07 to 0.31 ± 0.10) depending on the age of the animal, type of movement, if a human was in the animals’ sight line and whether the score was the observed score or the observed score converted to a categorical score [34]. When comparing the different methods of assessment, it was concluded that a categorical score had the highest heritability and was the most reliable indicator of temperament [34]. Behaviour when tethered, was reported to have a low heritability in German Angus (0.06 to 0.1 ± ≥0.06), and a moderate heritability (0.17 to 0.29 ± ≥0.12) in German Simmental calves [70]. This demonstrates the breed effect, however, this was a small study of between 192 and 271 cattle.

### 3.4. Genomics Background

Thirteen papers were allocated to the genomics review, three of these did not report any significant associations. Significant associations are listed in Supplementary Tables S3 and S4. No significant associations were found between the DRD4 fragment of chromosome 9 [58], the MAOA gene on chromosome X [76], the CRH (Chromosome 14) or LEP (Chromosome 4) [67] on animal behaviour. Eleven papers reported candidate genes and three reported associated SNPs with the traits of interest. Genetic associations with behavioural traits were found across the genome. None of the studies found a significant association with chromosome 13. The highest number of detected genes (nine) was on chromosome 9, however, six of these were for milking temperament and from the same study [64]. The other traits with associated genes were temperament and habituation [35] and habituation measured through vocalisation [69]. Milking temperament was measured subjectively by farmers within the first six months of milking in a first parity cow. Milking is carried out every day, and so there is a potential for cows to become habituated to the procedure and adapt their behaviour. It has been reported that 10 days of training can reduce the stepping and kicking behaviour of first parity cows in the parlour [77]. Different genes were reported by the studies, and without clarification on the stage within the first six months that the temperament assessment was carried out the milking temperament and habituation behaviours cannot be linked. Two SNPs on chromosome 9 (rs109313646 and rs111019360) were found to be associated with the duration of exploration of a novel object [66], one of which (rs111019360) was within 100 kb of the LOC781799 gene.

Milking temperament also had multiple gene associations on chromosome 27, however, the seven reported associations were all from the same study previously detailed [64]. This study also reported associations between chromosome 6 with milking temperament. Five further associations on chromosome 6 were found with aggression during gestation [51], habituation measured through walking, escape running events during socialisation [69], and FS [59]. An SNP association was reported on chromosome 6 for exploration in an open field test. Six gene associations were reported on chromosome 1, three of these were for traits which were related to habituation [35,69], two were milking temperament [64] and one for Temperament [58]. SNP rs41255467 on chromosome 1 was associated with FS and temperament score [36]. Five associations were found on chromosome 29, three of these were from the same study, for habituation to social separation measured through vocalisation and two consecutive measures of flight distance [69], and one was for temperament [69] and the remaining association was for milking temperament [64]. There were three or fewer associations on all other chromosomes other than chromosome 13 which had no reported associations. The greatest number of associations was reported for milking temperament, with the majority of associations reported from one study [64]. This study used a whole genome imputed model, whereas other studies targeted specific areas of the genome. This demonstrates the importance of technological advances and using high-resolution, modern techniques.

This spread of associations across the genome is attributed to the behaviour being influenced by multiple genes [51]. Many behavioural traits were found to be independent measures measuring different characteristics of the animal [57]. This may explain why they are controlled by different areas in the genome. Other variations in the associations can be attributed to the different ethological tests used to evaluate the behavioural traits [69]. Different associations were found with different breeds, and it was reported that Japanese black cattle might have a different polymorphism affecting temperament than Western breeds [67]. In addition to the variation in behavioural measures, cattle breeds and genetic assessment, the numbers of animals included in each study varied, ranging from 61 [67] to 4381 [64]. The number of animals was generally limited by the availability of recorded phenotypic data, with studies of ≥1000 animals depending on the automated recording of FS [59] or farmer-reported rather than standardised subjective scores [43,51,64]. More reliability would be provided by studies which include accurate phenotypic data from a high number of animals.

### 3.5. Production Traits Related to Behaviour

Twenty four studies reported the correlations or associations between production and behavioural traits. It is difficult to assess from the correlation coefficient whether a relationship is favourable or not as some studies had an inverse scale for behavioural traits and some did not. For the purposes of this review, it is stated that a study is ‘favourable’ if animals with a better temperament performed better. The correlation can be between 0, no relationship and 1 (or −1), a perfect relationship. This paper considers a correlation of 0 to 0.10 as no clear correlation, 0.11 to 0.30 as weak, 0.31 to 0.80 as moderate and 0.81 or greater as a strong correlation. Where correlation coefficient is not reported, the category of the relationship was derived from the text in the original study. Three groups of production traits (Supplementary Table S5) were considered and discussed in the next paragraphs.

#### 3.5.1. Intake, Bodyweight and Growth

A principle components analysis found that the calf’s personality traits would dictate its feeding behaviour, with more curious calves starting to consume starter diets at an earlier age [68]. Animals with a lower DMI were reported to be calmer [57], with weak favourable relationships reported [60,62]. Residual Feed Intake (RFI) measures an animal’s efficiency independent of growth [78]. Both genetic and phenotypic RFI was found to have a favourable, moderate correlation with FS [62], however, no clear relationship was found between FS and RFI in a study by [60]. Feed conversion was found to have a favourable, weak correlation with FS, however, the feed conversion rate was found to be unfavourably linked to FS [62].

Docility scores were found to have no clear correlation with body weight at 200 days or 400 days of age in Angus and Limousin cattle [49], or with yearling weight of Limousin heifers [50]. Conversely, a weak favourable association was found between yearling weight and aggression in Limousin heifers [50]. Favourable, weak relationships between both weaning weight and yearling weight with CS were reported in Hereford cattle [52] and between body weight and FS [60]. Highly significant favourable associations between avoidance distance and both 120-day and 140-day weights of breeding Limousin bulls were reported by [71]. A favourable moderate relationship was found between final body weight and FS [62].

Associations between growth traits and behavioural traits ranged from no correlation to moderately favourable. Growth efficiency was reported to be associated favourable and moderately with FS [62]. Whereas objective CS was found to have no clear correlation with Average Daily Gain (ADG), a significant, favourable relationship between ADG during the fattening period and CS was reported in beef cattle [54]. Chute score was not found to be associated with ADG in German Angus cattle [53], however, favourable relationships were found in the same study between ADG and CS of Charolais cattle (weak relationship) and moderate and favourable relationships between ADG and CS in Simmental, Hereford and Limousin cattle [53]. Similarly, favourable weak relationships were found between ADG and FS in German Angus, Charolais and Limousin with the relationship found to be favourable and moderate in Hereford cattle [53]; demonstrating the variation in behavioural traits between breeds. This variation in breeds is further demonstrated in a study by Gauly, Mathiak and Erhardt [70]. Although no clear correlation was found between German Angus docility prior to handling and ADG, favourable, weak associations were reported between German Angus docility during handling, and Simmental docility both prior to and during handling with their ADG [70]. Other studies of FS and ADG found no clear correlation [60] or weak, favourable associations [62].

#### 3.5.2. Carcass Traits

A favourable relationship was reported between carcass weight and FS, however, this was not found to be significant [55], a significant favourable association was reported between isolation score and carcass weight, with favourable, moderate associations between Temperament and Carcass weight reported by [38]. Despite the favourable relationship between carcass weight and temperament, an unfavourable relationship was reported between temperament and Yield estimate [38].

Unfavourable relationships between rib eye area, subcutaneous fat thickness and temperament [38]; tenderness and isolation score [54]; ultrasound longissimus area and ultrasound back fat with FS [62] were reported. No clear correlations were reported between Ultrasound carcass analysis and docility [49], marbling and temperament [38], marbling and CS [52] marbling and disposition [47], or tenderness assessed by Warner Bratler Shear Force and FS [33]. Despite this, favourable relationships between LLM area and FS [55], marbling and FS [55,62]. Moderate associations between rib thickness and temperament were also reported [38]. This demonstrates that the correlations are population specific, and to understand potential links with production and behavioural traits it will be necessary to conduct a study of the population of interest.

#### 3.5.3. Fertility and Milk Production

For a cow to be considered fertile she must be able to get in calf, gestate the calf to full term and deliver a live calf. The cow must then be able to produce sufficient quality and quantity of milk to sustain her calf or for a dairy enterprise. In order to get into the calf, a heifer must reach sexual maturity. It was reported that both docility and aggression had unfavourable associations with age at puberty, whereby aggressive and/or less docile heifers reached puberty earlier [50]. Although temperament was not found to influence the number of inseminations per gestation [79] or the number of cows in-calf during the first six weeks of the mating period [80], a moderate favourable relationship was reported between insemination rate and temperament [80]. Once cows were in-calf, there was found to be no clear correlation between gestation length and docility [49].

A small pelvic opening can lead to dystocia which is dangerous for both cow and calf [81]. It was reported that aggression and docility had favourable correlations with a pelvic opening but aggression had a stronger correlation [50], thus calving may be easier for calmer animals. This is reflected in the favourable weak associations between behavioural traits (docility and aggression) with calving ease, in a study of Limousin heifers [50]. A productive cow will have multiple calvings throughout its life, and as such requires to come on heat and be mated post calving. Reducing the days between parturitions or calving interval (CI) can be used as a measure of fertility in cows [82], no clear correlation between CI and temperament [80]. Days to first heat were found to have no correlation with aggressiveness, but unfavourable weak correlations with temperament and milking temperament [42]. No correlation with the time between calving and first service first service post-calving was reported [80]. Survival, defined as the maximum number of calvings by a cow, did not have a clear association with docility in a study of Pirenaica cattle, however, a tendency suggested further investigation of the potential relationship should be carried out [41]. Survival has alternatively been described as survival to the next calving [80] and found to have a moderate, favourable association with temperament. Although not described in the studies, it is possible that a strict culling policy of poor temperament animals may have affected this.

Behavioural reactivity of dual-purpose Simmental cows significantly influenced milk yield and composition, with calmer cows having a higher milk yield [79]. Nervous animals were found to have higher fat and protein percentages, however, this was attributed to the lower milk yield from these animals [79]. Milk yield had no correlation with docility, and a weak favourable correlation with aggressiveness [50]. Fear of humans accounted for 19% of the variation in milk yield between farms, with farms with more fearful animals having a lower milk yield [65], conversely, one study reported that excitable primiparous cows had a higher daily milk yield and lactation yield than calmer cows [83]. The study noted that there was little supporting evidence for this from other studies and this was attributed to methodological differences between studies [83]. In addition to milk yield, behavioural traits were found to be associated with management traits related to milking. Milk leakage is a negative trait which can lead to bacterial infection [84], temperament was found to have negative correlations with milk leakage in both manual milking systems and AMS [74]. Milking speed is an important trait as it can disrupt the throughput of cows in a parlour and increase the time required to milk the herd [63]. Milking temperament was reported to have a weak, favourable correlation with milking speed with cows with better milking temperament letting down milk quicker [63]. This is supported by small, positive correlations with milking speed and temperament in both manual milking systems and AMS reported in other studies [74], however a study of Brown Swiss cattle found unfavourable to no correlation between behavioural traits and milking speed [42]. High genetic correlations were found between teat cup attachment failures and temperament of Swedish Holstein and Swedish Red dairy cows in AMS, with calmer cows having fewer failures [39]. This is supported by improved connection time and attachments in cows with better farmer-assessed temperament [44], however, the handling temperament had a weaker relationship with attachments and no correlation with attachment time.

### 3.6. Limitations of the Review and Search Protocol

The study discarded one paper which was not published in English. This paper had an English abstract which appeared to support the other studies included in the review. However, the wide range of studies included which were in general agreement suggest that the coverage of the review was adequate.

Some environmental aspects are taken into account through the analyses of automatic and conventional milking or the use of embryo transfer to remove parental effects on temperament. However, to direct improvement of temperament and animal behaviour it is necessary to take a multifaceted approach which considers environmental and genetic effects.

There were varying results for different behavioural measures in different studies but also within the same study [54]. Although this could not be conclusively explained, it was attributed to either type 1 errors or temperament traits varying by age and so focusing on a specific time in the animal’s life was more likely to identify an association [54]. The variation in behavioural measures means that it is difficult to conclusively link the measures used in a controlled environment to behaviour on the farm due to the variation in farm management systems, thus to direct policy on a specific population of cattle, it is necessary to carry out a targeted study of that population. Additionally, many of the studies included in the review had small sample sizes (*n* < 1000) which limits their reliability and how they can be applied to novel populations.

## 4. Conclusions

A search protocol to return studies which described the heritability, potential associations with production or the genomic areas of interest of behavioural traits in *Bos taurus* cattle was developed and returned 2238 papers. Relevant papers were assigned non-exclusively to groups of heritability (22), genomic associations (13) and production traits related to behaviour (24). The heritability of behavioural traits means that additive improvement of temperament is possible and beneficial to animal producers. Correlations between temperament and production traits suggested that selection against animals that are highly reactive to improve welfare and ease of handling would not have detrimental impacts on productivity and reproductive outputs. Although there were genes and SNPs reported, the breed and animal type differences between studies meant that a meta-analysis was not possible. The findings of this paper can inform breeding decisions and if employed with suitable management lead to the improvement of animal behaviour and, in turn, farm safety. Furthermore, the variation in heritability estimates, correlations with production and genomic associations of behavioural traits reported in the studies reviewed highlights that these relationships are population specific. There was also found to be variation in management systems and the methods of behaviour assessment and scoring. Thus, to accurately assess the heritability, genetic associations with production and genomic areas of interest for behavioural traits, a large-scale study of the population of interest using uniform, comparable methods would need to be required.

## Figures and Tables

**Table 1:** The Population, Exposure, Outcome (PEO) framework of terms used in the literature search; the search algorithm was designed using the Boolean operators “AND” & “OR” to ensure that at least one term from each PEO component was returned.

Population (Bovine)	Exposure (Genetics)	Outcome (Behavioural)	
Cattle,	QTL,	Temperament,	Docility,
Cow,	“Gene”,	Aggressi *,	Fear *,
“Steer”,	Genetic *,	“Chute Score”,	“Crush Score”,
Heifer,	Heritab *,	Flight,	“Exit Time”,
Bull,	Marker,	“Exit Speed”,	“Exit Score”,
Dairy,	SNP,	“Exit Velocity”,	Excit *,
Beef,	GWAS,	“Movement Measuring Device”,	
Herd,	Genomic *	“Strain Gauge”,	“Coping Style”,
“*Bos taurus*”		Boldness,	Personality,
		Proactive,	Reactive

* Indicates the term is truncated and the ‘wild card’ can represent any character(s); inverted commas indicate that the exact term must be matched.

**Table 2:** Behavioural traits (grouped by trait category in italics) which were considered to have reported high accuracy heritabilities (SE ≤ 0.05).

Trait	Heritability ± SE	Breed	*n*	Age at Test	Reference
*Temperament, disposition and/or docility*					
Temperament (CMS) *	0.09 (0.01)	Dairy breeds	1,872,979	First parity cows	[74]
Farmer assessed temperament	0.10 (0.01)	Holstein	126,614	Mature cows	[44]
Temperament (AMS) ^†^	0.05 (0.01)	Dairy breeds	72,683	First parity cows	[74]
Handling temperament	0.11 (0.02)	Holstein	8108	Mature cows	[44]
Temperament	0.11–0.12 (≤0.05)	Japanese black	7897	6–9 months	[38]
Temperament	0.26 (0.01)	Dairy	843	Heifers	[75]
Docility	0.19 (0.03)	Pirenaica	2412	Mature cows	[41]
Docility	0.29–0.38 (≤0.03)	Limousin	23,453	Weaning	[48]
Docility	0.21 (<0.01)	Angus	50,935		[49]
Docility	0.46 (<0.01)	Limousin	50,930		[49]
*Chute score*					
Chute score	0.29 (0.02)	Hereford	25,037	Weaning	[52]
*Aggression*					
During separation	0 (0.05)	Simmental	140	Heifers	[37]
During gestation	0.06 (0.02)	Charolais	5954	Mature cows	[45]
At parturition	0.19 (0.05)	Charolais	5881	Mature cows	[45]
Aggressiveness	0.08 (0.02)	Limousin	2781	Heifers	[50]
*Milking temperament*					
Milking temperament	0.13 (0.01)	Dairy cows	1,940,092	First lactation	[63]
Milking temperament	0.04 (0.04)	Brown Swiss	2259	Mature cows	[42,43]

* Conventional Milking System; ^†^ Automatic Milking System

## Data Availability

Not applicable.

## Supplementary Materials

The following supporting information can be downloaded at: https://www.mdpi.com/article/10.3390/ani12192602/s1, Table S1: Traits described in the studies returned by the search criteria and how they were scored and where they were undertaken; Table S2: Behavioural traits which were considered to have low accuracy heritabilities (SE > 0.05); Table S3: Gene associations reported in studies returned by the search criteria; Table S4: Single Nucleotide Polymorphisms (SNP) which were found to have associations with behavioural traits in studies returned by the search criteria; Table S5: Relationship between behavioural traits and production traits reported
